# A Role for Toll-Like Receptor Mediated Signals in Neutrophils in the Pathogenesis of the Anti-Phospholipid Syndrome

**DOI:** 10.1371/journal.pone.0042176

**Published:** 2012-07-31

**Authors:** Gerd Gladigau, Philipp Haselmayer, Inge Scharrer, Markus Munder, Nadine Prinz, Karl Lackner, Hansjörg Schild, Pamela Stein, Markus P. Radsak

**Affiliations:** 1 Institute of Immunology, Johannes Gutenberg-University Medical Center, Mainz, Germany; 2 Third Department of Medicine, Johannes Gutenberg-University Medical Center, Mainz, Germany; 3 Institute of Clinical Chemistry and Laboratory Medicine, Johannes Gutenberg-University Medical Center, Mainz, Germany; Leiden University Medical Center, Netherlands

## Abstract

The anti-phospholipid syndrome (APS) is characterized by recurrent thrombosis and occurrence of anti-phospholipid antibodies (aPL). aPL are necessary, but not sufficient for the clinical manifestations of APS. Growing evidence suggests a role of innate immune cells, in particular polymorphonuclear neutrophils (PMN) and Toll-like receptors (TLR) to be additionally involved. aPL activate endothelial cells and monocytes through a TLR4-dependent signalling pathway. Whether this is also relevant for PMN in a similar way is currently not known. To address this issue, we used purified PMN from healthy donors and stimulated them in the presence or absence of human monoclonal aPL and the TLR4 agonist LPS monitoring neutrophil effector functions, namely the oxidative burst, phagocytosis, L-Selectin shedding and IL-8 production. aPL alone were only able to induce minor activation of PMN effector functions at high concentrations. However, in the additional presence of LPS the activation threshold was markedly lower indicating a synergistic activation pathway of aPL and TLR in PMN. In summary, our results indicate that PMN effector functions are directly activated by aPL and boosted by the additional presence of microbial products. This highlights a role for PMN as important innate immune effector cells that contribute to the pathophysiology of APS.

## Introduction

The anti-phospholipid syndrome (APS) is a systemic autoimmune disease characterized by an adaptive immune response against self membrane anionic phospholipids or associated plasma proteins resulting in the generation of anti-phospholipid specific antibodies (aPL) [1] and APS patients show a high risk for venous or arterial thrombosis. 2% of the general population develop APS affecting in particular females [2], [3]. For women, the presence of aPL is a risk factor associated with pregnancy complications and loss [4]. Although APS is considered as an autoantibody-mediated disease, there is growing evidence that aPL are necessary but not sufficient for the clinical manifestations of the syndrome. In particular, mediators of innate immunity are increasingly recognized to be additionally involved. Analyzing the participation of aPL in pregnancy loss during APS in more detail revealed that aPL apparently have a direct impact on complement activation as shown in animal models, where LPS pretreated rats received transfer of polyclonal IgG aPL from patients with APS [5]. Thrombus is induced dependent on the activation of C5 and C6 as well as on β2-GPI-reactive aPL. In line with these results, Girardi et al. showes that C5-deficient mice are protected from aPL-induced pregnancy loss [6]. In this context, the interaction of C5a with the C5a receptor induces the activation of polymorphonuclear leukocytes (PMN) and leads to the generation of reactive oxygen species (ROS) and release of granular components. Depleting PMN in the presence of aPL avoids fetal resorption [6]. Therefore PMN seem to be key players in aPL-induced pregnancy loss. As an underlying mechanism, PMN express tissue factor (TF) upon C5a-induced activation [7] which is an important contributor to neutrophil-mediated fetal injury and loss [8]. Altogether, these findings suggest a role for innate immunity in APS pathogenesis. Recently, also Toll-like receptor (TLR) mediated signals have been implicated in the activation cascade of aPL induced thrombus formation. There is evidence that TLR4 [9] and the signalling cascade via MyD88 [10] contribute to the phenotype of APS. Furthermore, the participation of other TLRs cannot be excluded as already shown for TLR7 on plasmacytoid dendritic cells and TLR8 on monocytes [11], [12]. PMN might directly act as stimulators of APS phenotype since they constitutively express various TLRs.

To address this question, we analyzed the impact of a purified human monoclonal aPL [11], [13], [14] on the activation of the PMN. We found that aPL alone were only able to induce minor activation of PMN effector functions. However, in the additional presence of LPS or Pam_3_Cys the activation threshold was markedly lowered indicating a synergistic activation pathway of aPL and microbial products also in PMN. These results suggest that PMN as important innate immune effector cells are directly activated by aPL under inflammatory conditions and therefore may be an important contributor to the pathophysiology of APS.

## Materials and Methods

### PMN Purification

Citrated blood of healthy volunteer donors was purified with Polymorphprep (Progen, Heidelberg, Germany) using an established protocol [15]. Briefly, 10 ml of citrated whole blood was layered over the Polymorphprep gradient in a 1∶1 ratio. After centrifugation PMN were extracted harvesting interphase. After hypotonic lysis step cells were incubated and stimulated as indicated. All human studies were performed after obtaining written consent from healthy volunteer donors in accordance with the Declaration of Helsinki and were approved by the Landesaerztekammer Rhineland-Palatine Ethics Committee according to the institutional guidelines.

### Stimulation of PMN

Lipopolysaccharide (LPS) from *Salmonella typhimurium* (Sigma Aldrich, Taufkirchen, Germany) was utilized in a final concentration of 100 ng/ml or titrated (1 ng/ml, 10 ng/ml, 100 ng/ml) as indicated. Titrated concentrations (10 µg/ml, 1 µg/ml, 100 ng/ml) of Palmitoyl-3-Cys-Ser-(Lys)_4_ (Pam_3_Cys, EMC Microcollections, Tübingen, Germany) were employed as TLR2 ligand. Recombinant human lipopolysaccharide binding protein (LBP, RayBiotech, Norcross, USA) was utilized in a final concentration of 10 ng/ml. Human monoclonal aPL (clones HL5B and HL7G) were utilized in a final concentration of 10 µg/ml and have been described previously [11], [13], [14]. Human control IgG (Sigma Aldrich) was also used in a final concentration of 10 µg/ml. Human monoclonal aPL and human control IgG were both endotoxin-free as tested with limulus amebocyte lysate assay.

### FACS-staining of PMN

PMN were incubated with L-Selectin (CD62L)-APC or CD11b-PE in PBS with 1% BSA and 0.05% sodium azide (Sigma-Aldrich; FACS buffer) on ice. To remove unbound mAbs, cells were washed twice with FACS buffer. All FACS-analyses were performed with a FACSCanto or a LSRII Flow Cytometer and FACSDiva software (BD Pharmingen, Hamburg, Germany).

### Analysis of the Oxidative Burst

Hydrogen peroxide activity was detected using oxidation of dichlorofluorescein diacetate (DCFH-DA, Sigma Aldrich) to DCF shown in green fluorescence. Kinetics were measured with a fluorescence reader (SpectraFluor 4 or Genios, Crailsheim, Germany) as described previously [15]. Specific fluorescence index (SFI) of stimulated cells was assessed subtracting fluorescence of labelled cells incubated with medium alone. In some Figures, fold induction of stimulated cells was calculated relative to cells incubated with medium alone.

### Detection of IL-8 Release

For detection of IL-8 release supernatants of PMN culture were harvested after 2 or 6 hours of stimulation as indicated and analyzed by enzyme-linked immunosorbent assay (ELISA, R&D Systems, Wiesbaden, Germany) according to the manufacturer’s instruction.

### Detection of Phagocytosis

The phagocytic activity was evaluated by ingestion of PE-labelled polystyrene microspheres (diameter 1 µm, Fluoresbrite Plain Microspheres PCRed, Polysciences, Warrington, PA) as described previously [15]. Briefly, PMN (2×10^5^ per well) were stimulated as indicated in the presence of 5×10^6^ microbeads for 90 min at 37°, then kept on ice, washed twice in FACS buffer, and fixed in 1% paraformaldehyde in PBS. Analysis was performed using FACS. The amount of phagocytosis was quantified as percentage of PE-positive events in the PMN region as defined by light scattergram.

### Detection of Apoptosis

Apoptotic cells were quantified by detection of DNA fragmentation, evaluating hypodiploid nuclei according to a modified protocol described by Nicoletti *et al*. [16]. Cells (2×10^5^ per well) were resuspended in staining buffer (50 µg/ml propidium iodide in 0.1% sodium citrate and 0.1% Triton X-100, all from Sigma Aldrich), incubated for 2 hr at 4°C and analysed by FACS.

### Statistical Analyses

Statistical analyses of the data were performed Tukey’s test. *P* values less than 0.05 were considered statistically significant.

## Results

### aPL Prime the TLR4 Induced Activation of the PMN Oxidative Burst

PMN are the primary innate immune effector cells combating pathogenic microorganisms, such as bacteria and fungi. Inflammatory signals, i. e. IL-1 or TNF-α, or the recognition of microbial patterns prime the activation of the NADPH oxidase and induction of oxidative burst. This leads to the formation of toxic reactive oxygen species (ROS) [17], [18], that mediate microbial killing and adversely also cause tissue damage [19] that is responsible for fetal loss or injury during pregnancy in the context of APS [8].

**Figure 1:**
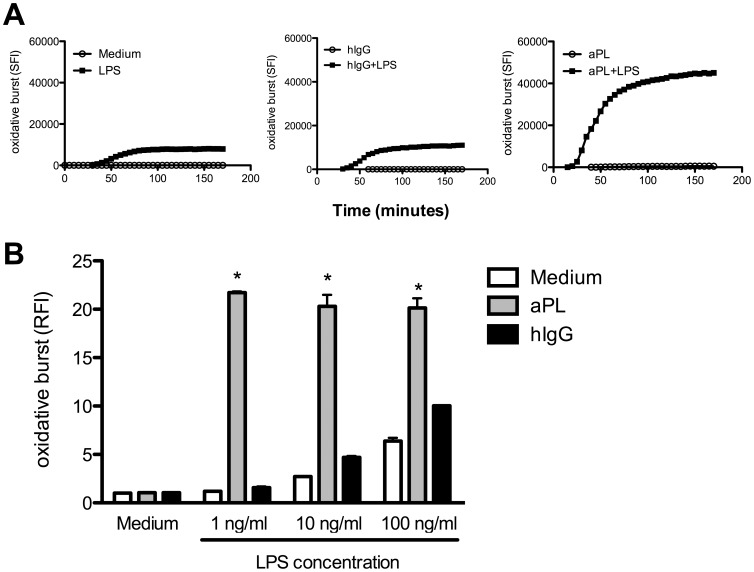
aPL increase oxidative burst activity in human PMN. Human PMN (2×10^5^ cells per well) were incubated with medium or LPS in the presence or absence of aPL or human IgG (10 µg/ml). Production of radical oxygen species was determined by oxidation of dichlorofluorescein diacetate to DCF and measured in a fluorescence reading device. A) Detection of specific fluorescence index (SFI) over time was calculated by subtraction of the background fluorescence of labelled cells incubated in medium. LPS stimulation was performed using 100 ng/ml. B) Titration of LPS concentrations (1 ng/ml, 10 ng/ml, 100 ng/ml) as indicated was analysed after 170 minutes as fold induction. Data shown are from one representative experiment out of 3 independent with 2 replicates per group. *Indicates significant difference of aPL treated groups compared to corresponding hIgG controls (p<0.05 in a Tukey’s multiple comparison test).

To investigate the direct influence of aPL on PMN, we incubated purified PMN with a human monoclonal aPL *in vitro* and monitored ROS formation over time. To assess an optimal experimental setup, we first titrated aPL and chose 10 µg/ml as the final concentration utilized in all experiments (see Figure S1). As shown in Figure 1A (open circles), aPL alone was unable to induce oxidative burst activity, comparable to human control IgG as well as the medium control.

Never the less, there are synergistic activation pathways in PMN, i. e. mediated by innate immunoreceptors that synergize with TLR mediated activation. This prompted us to investigate the influence of aPL in the additional presence of the TLR4 ligand LPS, as a prototypic TLR stimulus in the context of Gram-negative infections. As furthermore shown in Fig. 1A (filled squares), LPS alone induced the oxidative burst in a comparable way as in the hIgG control (compare left and middle panel), indicating that the amount of hIgG present in the culture does not induce oxidative burst activity in a non specific, i. e. Fc receptor mediated manner. In contrast, the ROS production in the presence of aPL was greatly enhanced (right panel) indicating that aPL facilitates TLR4 induced ROS production by a synergistic activation mechanism.

To evaluate this effect in more detail, we titrated the amount of TLR ligand. As demonstrated in Figure 1B, the oxidative burst activity was dependent on the concentration of LPS. While LPS at low concentrations (1 ng/ml) alone was unable to induce detectable oxidative burst activity, the additional presence of aPL still showed strong induction of the ROS production further underpinning a truly synergistic activation pathway. These results suggest that aPL directly interact with PMN, maybe amplifying inflammatory responses induced by danger signals such as the TLR4 agonist LPS.

Many inflammatory stimuli have an influence on PMN survival, either enhancing or delaying PMN apoptosis. To this end we stimulated purified PMN with LPS in the presence or absence of aPL and analysed quantity of apoptotic cells via FACS. The incubation of PMN with aPL showed an apoptosis rate similar to the medium control. The stimulation of PMN with LPS alone significantly reduced quantity of apoptotic cells. As expected from the aPL alone data, the combinatory treatment with aPL and LPS had no further impact on cell viability compared to LPS stimulation (see Figure S2).

These results indicate, that the activation status mediated by the treatment with aPL are independent of cell viability and PMN survival is only influenced by TLR stimulation via LPS.

### Independent and Cooperative PMN Stimulation for Phagocytosis and CD11b-upregulation with aPL and LPS

**Figure 2:**
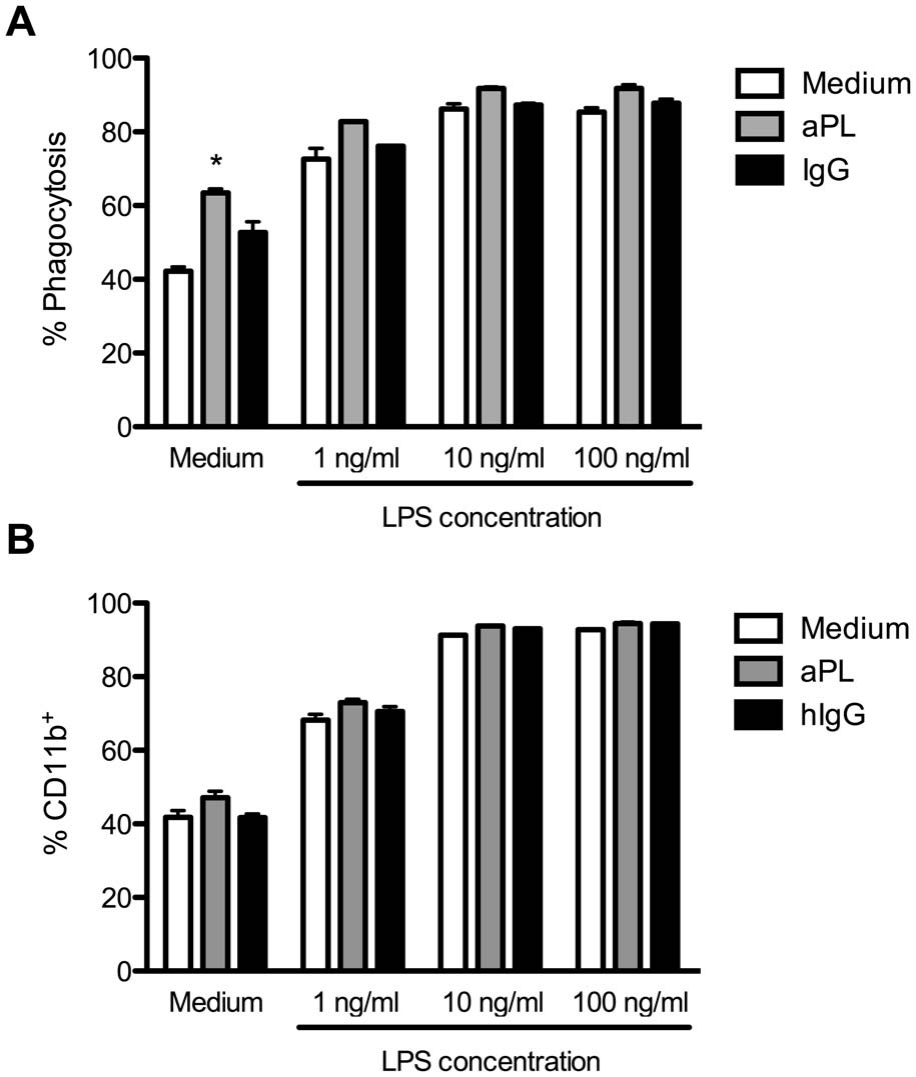
aPL stimulate PMN for phagocytosis. Human PMN (2×10^5^ cells per well) were stimulated with medium, aPL or hIgG in the presence or absence of titrated LPS as indicated and analyzed by FACS. A) For quantification of phagocytic activity PE-labelled microspheres were added to the culture. B) Expression of CD11b was determined via surface staining. Data from one representative experiment out of 3 independent with two replicates as percent phagocytosis or percent CD11b^+^ cells is shown. *Indicates significant difference of LPS-stimulated groups compared to corresponding hIgG controls (p<0.05 in a Tukey’s multiple comparison test).

In addition to the oxidative burst with ROS formation, PMN are professional phagocytic cells by their ability to rapidly ingest opsonized particles. Since aPL alone did not induce oxidative burst activity in PMN, but acted in a synergistic way together with LPS, we further asked for the influence of aPL on another PMN effector function, namely phagocytic activity. To this end, we stimulated purified PMN as before, with titrated LPS concentrations, and evaluated the phagocytic activity of these cells using fluorochrome labelled polystyrene beads that are taken up by PMN and can be detected by FACS. As demonstrated in Figure 2a, unstimulated cells (42.2%, +/− 1.1%) as well as the cells treated with human control IgG (52.6%, +/− 2.9%) already showed phagocytic activity. However, the presence of aPL increased phagocytic activity as evidenced by enhanced uptake of PE-labelled beads (63.4%, +/− 1.1%). The treatment with LPS also led to a significant uptake of beads in a concentration dependent manner. This was slightly enhanced by the combinatory stimulation with aPL and LPS from 85.4%, +/− 1.1% without aPL to 91.8%, +/− 0.9% in the addition of aPL at the highest concentration of LPS.

This suggests that for aPL induced phagocytosis, such additional signals are not required to trigger this particular effector function, but can still be enhanced.

To further investigate in this point we analyzed CD11b expression upon stimulation of PMN, as a mediator of phagocytosis and cell-adhesion. As depicted in Figure 2b an upregulation of CD11b could be observed in a LPS-concentration dependent manner. The stimulation with aPL alone failed to induce a significant increased expression.

### aPL Induced Shedding of L-Selectin on PMN

**Figure 3:**
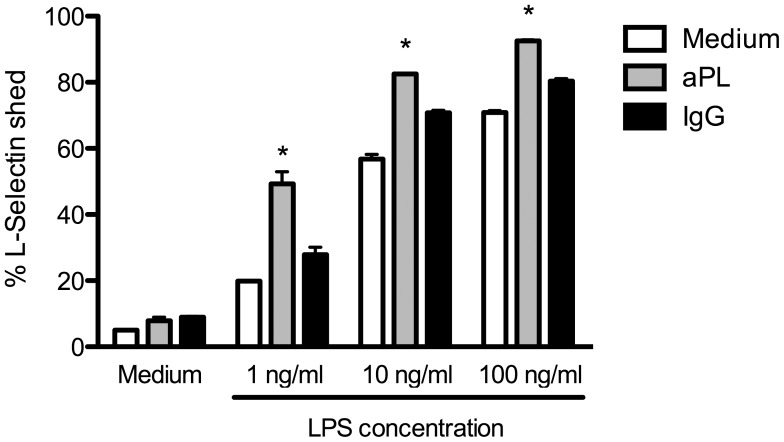
Synergistic induction of L-Selectin shedding mediated via aPL and LPS. PMN (2×10^5^ cells per well) were incubated with medium, aPL or hIgG in the presence or absence of titrated LPS. Shedding of L-Selectin was determined by surface staining of expressed molecules and analyzed by flow cytometry. Data shown are from one representative experiment out of 3 independent with 2 replicates per group. *Indicates significant difference of LPS-stimulated groups compared to corresponding hIgG controls (p<0.05 in a Tukey’s multiple comparison test).

For local control of infections, PMN need to migrate to inflamed tissues. In this context, they first loosely adhere to the vascular endothelium before firm attachment and rolling is initiated allowing the cells to transmigrate into the tissue. For the initial attachment of PMN to endothelial cells, L-Selectin (CD62L) expression level is important and activation induced shedding is widely used as a marker for PMN activation [20]. Therefore, we analyzed L-Selectin expression upon coincubation of PMN with aPL in the additional absence or presence of the TLR4 agonist LPS. As shown in Fig. 3, the presence of aPL does not induce L-Selectin shedding in PMN comparable to the medium or the hIgG control. In the additional presence of LPS, however, we observed a nearly complete loss of L-Selectin in a concentration dependent manner exceeding the effects of LPS alone.

This once again suggests a synergistic mode of activation for the shedding of L-Selectin.

### IL-8 Production by PMN is Induced Upon aPL and LPS Stimulation

**Figure 4:**
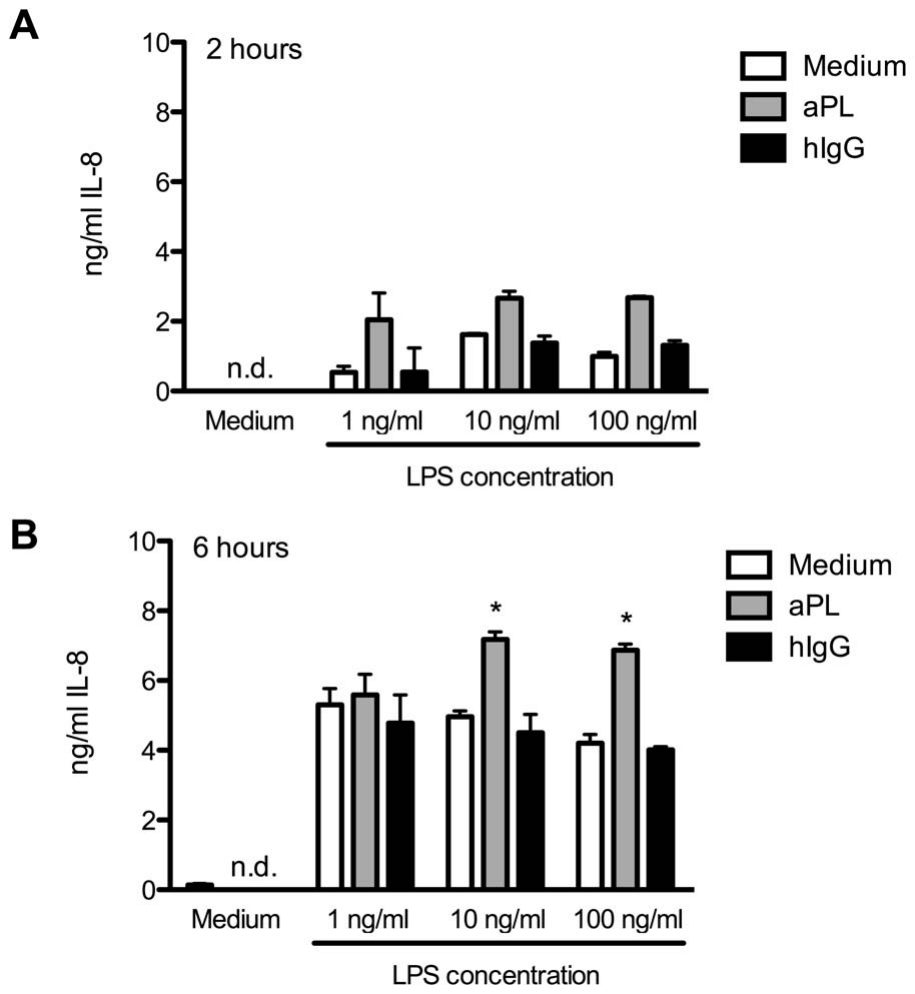
PMN release IL-8 upon stimulation with aPL and LPS. PMN (2×10^5^ cells per well) were incubated with medium, aPL or hIgG (10 µg/ml) in the presence or absence of LPS. Supernatants were harvested and an IL-8 specific ELISA was performed after A) 2 hours or B) 6 hours of incubation (37°C). Data shown are from one representative experiment out of 3 independent with 2 replicates per group. * Indicates significant difference of LPS-stimulated groups compared to corresponding hIgG controls (p<0.05 in a Tukey’s multiple comparison test).

Since PMN constitute the first line of defence against microbial and fungal pathogens, another hallmark function of PMN is the ability to recruit immune cells to a site of inflammation by the release of chemokines and cytokines. A major chemokine involved in the attraction of immune cells is IL-8 (also known as CXCL8). Thus an activated phenotype of PMN is characterized by the increased production and release of IL-8. Therefore we asked in a next step whether IL-8 is induced upon contact of PMN with aPL or the combination of LPS and aPL. To this end, we performed *in vitro* cultures of purified PMN and incubated them either with aPL, LPS or the combination and as a control with human IgG, as described before. After 2 or 6 hours of culture, supernatants were collected and tested in an IL-8 specific ELISA (Figure 4). As expected, the incubation of PMN with LPS induced activation of PMN shown in increased amounts of IL-8 compared to unstimulated or hIgG-treated cells after 2 hours (Fig. 4A) as well as after 6 hours (Fig. 4B). The contact of PMN with aPL alone does not induce IL-8 production. As shown before for the other effector functions, the combinatory stimulation with aPL and LPS again led to an additive activation depicted in further enhanced IL-8 production. This is already detectable at the lowest LPS concentration at an early time point, whereas at later time points the effect is transposed to higher LPS concentrations.

These data clearly demonstrate that the inflammatory cytokine IL-8 is produced and released in the presence of aPL and LPS.

### aPL and LBP Enhance Oxidative Burst in a Comparable Way

**Figure 5:**
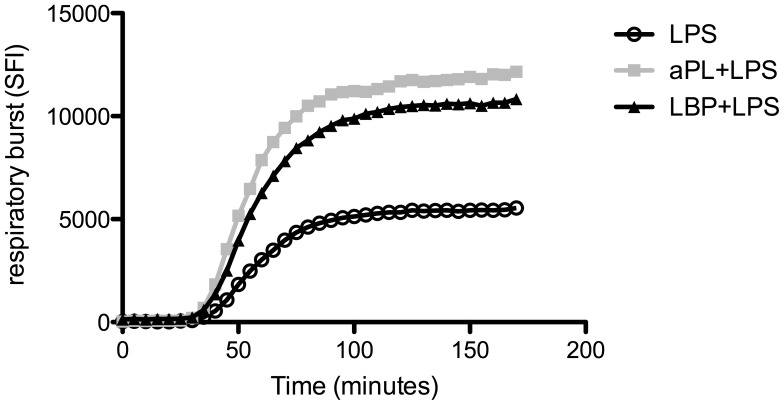
LPS stimulation can be enhanced by facilitating binding to CD14. Human PMN (2×10^5^ cells per well) were incubated with LPS (100 ng/ml) with or without the addition of aPL (10 µg/ml) or LPS-binding protein (LBP, 10 ng/ml). ROS formation was measured in a fluorescence reading device. Detection of specific fluorescence index (SFI) over time was calculated by subtraction of the background fluorescence of labelled cells incubated in medium. Data from one representative experiment with two replicates is shown.

Cells of the innate immune system make use of receptors recognizing common patterns of invading microorganisms. LPS as well as lipoproteins and others need to be bound and transported. Therefore the lipopolysaccharide-binding protein (LBP) and additional transport proteins participate as a first defence of the host immune response [21]. LBP is a secretory class 1 acute-phase protein and the synthesis beside the constitutive presence is induced via LPS, Gram-negative bacteria and agents such as turpentine [22]. After binding of LPS at the amphipathic lipid A moiety the processing and subsequent presentation is facilitated. The binding of LPS to membrane-bound CD14 is an essential step in initiating an innate immune response, which is catalyzed by LBP [21], [23] shown in experiments in a serum-free system, where the addition of LBP enhanced LPS-mediated stimulation of CD14^+^ cells 100- to 1000-fold [21], [24]. To evaluate LPS function in our *in vitro* PMN stimulation we incubated the purified cells with LPS in the presence or absence of LBP and additionally compared LBP+LPS with aPL+LPS stimulation. As depicted in Figure 5 LBP+LPS (black triangles) showed enhanced oxidative burst activity compared to LPS alone (open circles). The progress over time of LBP+LPS is congruent to aPL+LPS (grey boxes).

These results indicate that aPL may functionally play a similar role as LBP.

### aPL Treatment Together with TLR2 Ligation Synergistically Activate PMN

**Figure 6:**
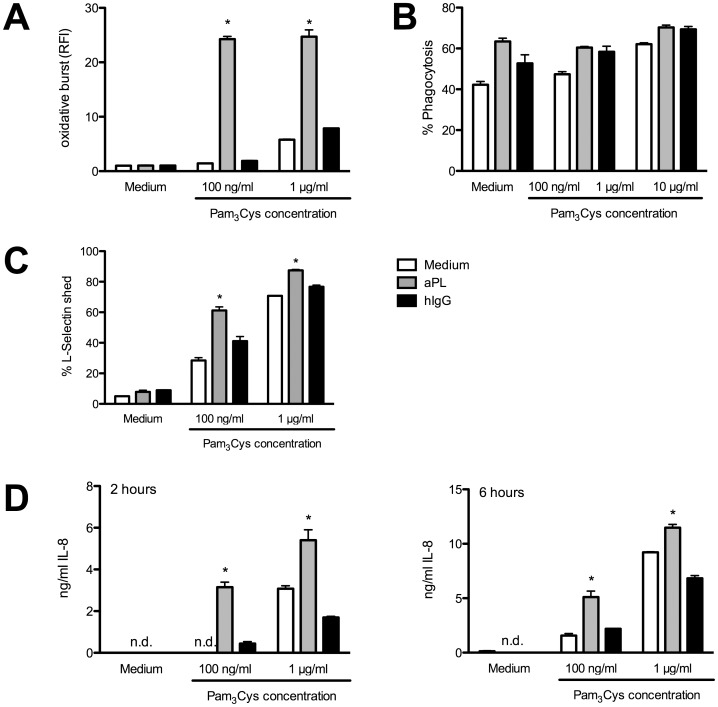
Synergistic activation of PMN with aPL and TLR2 ligation. Human PMN (2×10^5^ cells per well) were incubated with the TLR2 ligand Pam_3_Cys (100 ng/ml, 1 µg/ml, 10 µg/ml). A) Production of radical oxygen species was determined after 170 minutes. B) Phagocytosis rate was analysed defining percentage of PMN taken up PE-labelled microspheres. C) Shedding of L-Selectin was determined by surface staining of expressed molecules. D) Supernatants of stimulated PMN culture were harvested and an IL-8 specific ELISA was performed after 2 hours (left panel) or 6 hours (right panel) of incubation. Data shown are from one representative experiment out of 3 independent with 2 replicates per group. *Indicates significant difference of Pam_3_Cys-stimulated groups compared to corresponding hIgG controls (p<0.05 in a Tukey’s multiple comparison test).

The activation pathway via TLR4 illustrates the initiation of an immune response for invading Gram-negative microorganisms. Components of Gram-positiv bacteria are mostly recognized via TLR2 [25]. Ligands for TLR2 are bacterial lipopeptides, peptidoglycans and lipoteichoic acid. Neutrophils express most TLRs, including TLR4 and TLR2. Having analysed the role of TLR4-mediated signals in detail, we next focused on the impact of a different TLR ligation, namely TLR2, on PMN in the absence or presence of aPL. Analysing induction of ROS (Fig. 6a) revealed that the combination of aPL with Pam_3_Cys led to enhanced burst activity in the case of low amounts of TLR2 ligand (100 ng/ml and 1 µg/ml).

In the phagocytosis assay (Fig. 6b) aPL alone induced enhanced uptake of PE-labelled beads compared to hIgG or medium control. However, upon stimulation of PMN with aPL and various Pam_3_Cys concentrations we were unable to detect an additional or synergistic activity for this particular effector function.

The shedding of L-Selectin after stimulation of PMN displayed a synergistic activity after the combinatory incubation with Pam_3_Cys and aPL in a concentration dependent manner, depicted in Figure 6c. Consistent with the data described before, the release of IL-8 clearly shows synergistic capacities for Pam_3_Cys and aPL even at an early time point (2 hours) after stimulation (Fig. 6d).

Taken together these results demonstrate that aPL can enhance the stimulation of PMN mediated via different TLR ligands and is not only limited to LPS and TLR4 signalling pathways.

## Discussion

The APS is an acquired autoimmune disorder in which patients suffer from recurrent venous or arterial thrombosis or fetal loss. Although APS seems to be a major cause for pregnancy loss in otherwise healthy women [26], [27] and the presence of aPL support the induction of thrombus formation [28], [29], little is known concerning the direct and indirect impact of aPL and the participation of various cell types. There is evidence, that the presence of aPL is necessary for the manifestation of APS, but they need to be accompanied by additional factors [30]. Beside aspects like prolonged immobilization or surgical procedures, which themselves support thrombus formation, infections may also act as additional factors potentiating the impact of aPL [31]. In the context of innate immune activation, Toll-like receptors (TLR) will be an important cofactor. Direct recognition of microbial or viral products by TLR induces inflammatory responses that could contribute to the manifestation of APS. Since PMN are important participants of innate immune responses and express various TLRs [32], there might be a direct role for TLR mediated activation of PMN in the course of disease. PMN are well known to be involved in trophoblastic injury by complement activation and via the interaction of C5a with the C5a receptor [6] leading to PMN activation. In this model, the activation of PMN results as a second step in the signalling cascade of aPL without direct interaction. Our aim was to determine the direct influence of aPL on PMN and to evaluate the effects of an additional stimulus like TLR ligation on the PMN activation status. In our experiments, we show that human purified aPL had an impact on PMN function, namely on the induction of phagocytosis. In contrast oxidative burst activity, L-Selectin shedding as well as the release of IL-8 was not increased after aPL treatment when cells were incubated with aPL alone. Analyzing the impact of aPL in the presence of TLR4 ligand LPS or TLR2 ligand Pam_3_Cys demonstrates that additional activation of PMN together with aPL induced enhanced IL-8 production and L-Selectin shedding compared to aPL or LPS or Pam_3_Cys alone. In contrast, the oxidative burst induced by the combination of aPL and LPS or Pam_3_Cys was truly synergistic, exceeding the effects of LPS alone. This fits very well to the results of Redecha and coworkers who show an increased ROS production in PMN of aPL treated mice and a role for PMN derived ROS in oxidative trophoblastic injury and fetal loss [33]. In general, an upregulation of TLR4 on stimulated PMN either with aPL or LPS or the combination of both, could not be detected (data not shown), but is in line with already described data [34].

This synergistic activation pattern of aPL and TLR2/4 was not observed in the phagocytosis assay. Although, aPL induced some phagocytic activity in PMN, the effects of the TLR4 agonist LPS and the TLR2 agonist Pam_3_Cys were much more pronounced leaving no room for further enhancement of phagocytic activity even though ligands were titrated to very low amounts. A comparable effect of aPL on phagocytosis has been observed previously in mouse PMN [33]. Therefore our data extends experimental results from a mouse model to the activation of human cells. In addition, our results indicate that the effects of aPL on phagocytic activity of PMN is less pronounced than the effects induced by LPS or Pam_3_Cys – at least under the experimental conditions chosen. Nevertheless, since we used comparable amounts of LPS/Pam_3_Cys and aPL in the analysis of each effector function, it is safe to conclude that the effect of aPL on the activation of various PMN functions tested is distinct: While we find a strong but strictly TLR dependent activation of the oxidative burst, L-Selectin shedding and IL-8 release, phagocytic activity is only moderately activated and more or less overwhelmed by the additional TLR mediated activation signals. Concerning the synergism of aPL and LPS in ROS formation we could compare the observed results with those found while combining LPS and LBP. In both cases an increased oxidative burst activity could be detected and one could speculate for a comparable role of aPL and LBP enhancing the binding of LPS.

As pointed out, our data fit very well into the existing literature indicating the relevance of PMN and TLR in the context of APS [9], [33], [35]. However, the direct impact of TLR mediated signals on aPL activated PMN has not yet been assessed *in vivo* in animal models. Therefore, the biological impact of our observations is currently not clear and needs to be further validated in *in vivo* models.

One limitation of our studies concerns the use of monoclonal aPL. Although it has been isolated from an APS patient [11], [13], [14], we can not be sure that our results obtained with this monoclonal aPL is truly representative of typical polyclonal aPL directly from patients. On the other hand, only the use of a well-defined monoclonal aPL allows us to establish reliable experimental conditions to study the mechanisms of aPL induced cell activation. Further studies with hIgG fractions of APS patients will be needed to assess whether our experimental data also holds true with primary aPL.

Our experiments clearly demonstrate that aPL can directly act on immune cells, as shown here for PMN. Additional stimulatory signals for example via TLR in the course of microbial infections may enhance the inflammatory milieu thereby triggering the manifestation of APS. The participation of TLR7/8 in APS has already been shown for other cell types like monocytes and pDCs [11], [12]. Here the presence of aPL induced the production of TNF-a in monocytes which is dependent on TLR8. These data allow the conclusion that during APS complex signalling cascades are activated beginning with the formation of aPL that on the one hand activate the complement system, leading to subsequent activation of inflammatory cells; and on the other hand directly act on different cell types. Furthermore those reactions can be triggered by the presence of additional activating motifs e.g. TLR ligands during infections. Activating innate immune cells also leads to enhancing feedback loops via release of cytokines and the attraction of more inflammatory cells. Our data may pave the way to overcome current limitations analyzing the complex circumstances leading to the clinical manifestations of APS.

## Supporting Information

Figure S1 **Induction of oxidative burst is dependent on aPL concentration.** Human PMN (2×10^5^ cells per well) were incubated with LPS (100 ng/ml) together with medium or titrated hIgG or aPL (10 µg/ml, 1 µg/ml, 0.1 µg/ml). Detection of specific fluorescence index (SFI) over time was calculated by subtraction of the background fluorescence of labelled cells incubated in medium.
(TIFF)

Figure S2 **Stimulation of PMN with aPL has no impact on induction of apoptosis.** Human PMN (2×10^5^ cells per well) were incubated with LPS (100 ng/ml) with or without the addition of aPL or hIgG (10 µg/ml) Apoptotic cells were quantified by detection of DNA fragmentation via FACS. Data shown are from one representative experiment out of 3 independent with 2 replicates per group.
(TIFF)
