# Supplementary figures and images for: A Role for Toll-Like Receptor Mediated Signals in Neutrophils in the Pathogenesis of the Anti-Phospholipid Syndrome
Source: PLoS One. 2012 Jul 31;7(7):e42176. doi: 10.1371/journal.pone.0042176 (PMC3409186; doi:10.1371/journal.pone.0042176)

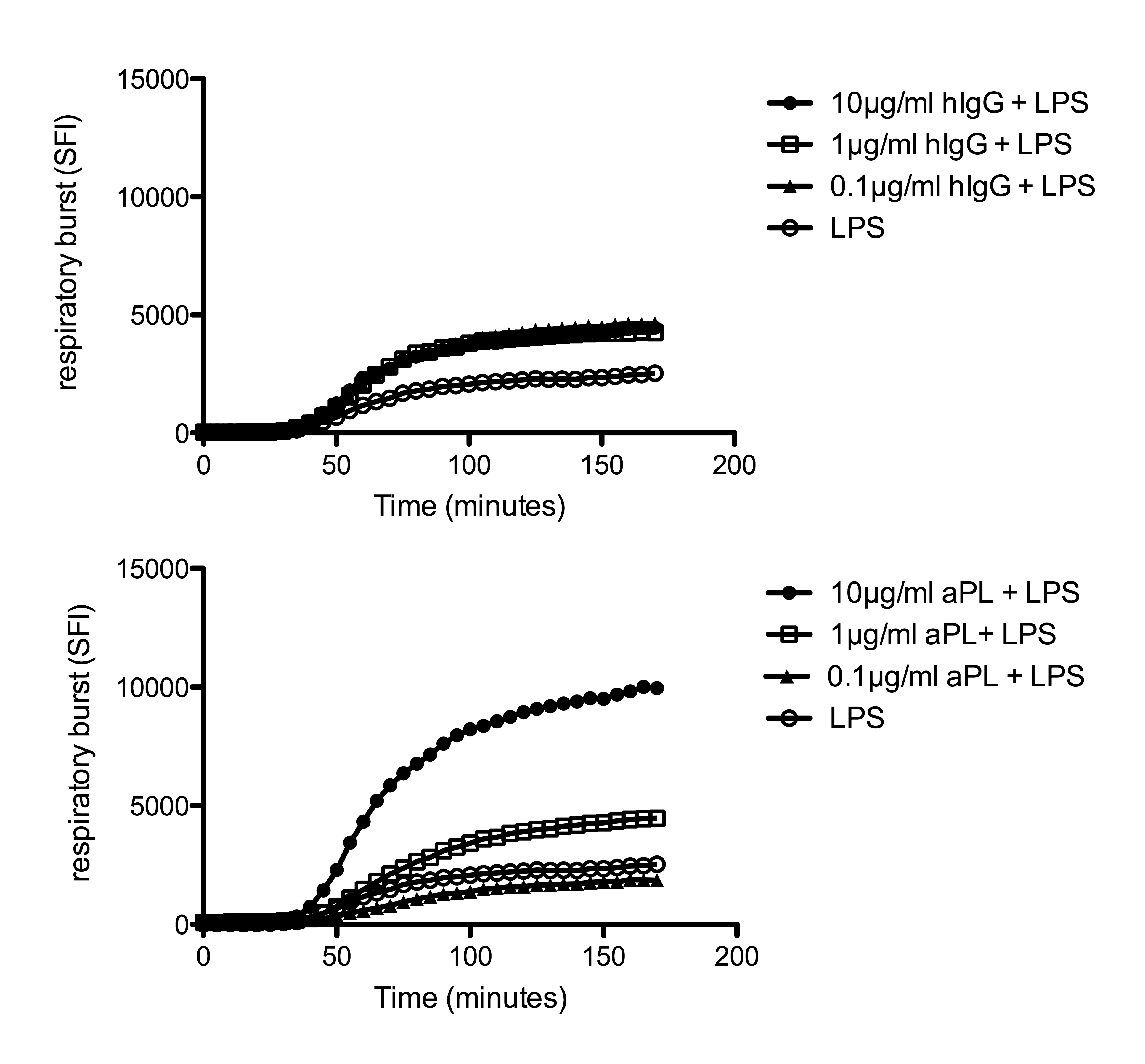

Supplement: Figure S1 — Induction of oxidative burst is dependent on aPL concentration. Human PMN (2×10^5^ cells per well) were incubated with LPS (100 ng/ml) together with medium or titrated hIgG or aPL (10 µg/ml, 1 µg/ml, 0.1 µg/ml). Detection of specific fluorescence index (SFI) over time was calculated by subtraction of the background fluorescence of labelled cells incubated in medium. (TIFF) [file pone.0042176.s001.tiff]

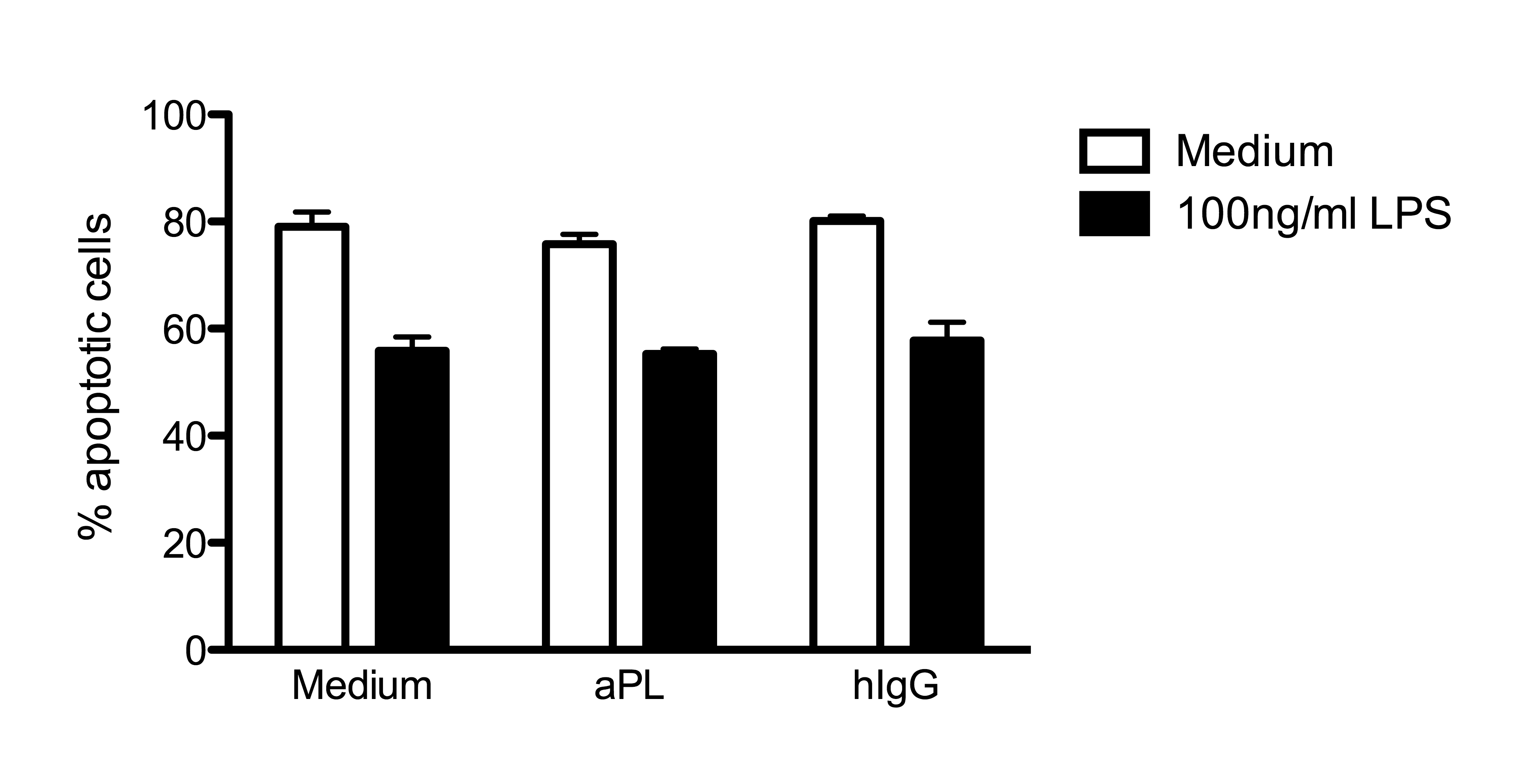

Supplement: Figure S2 — Stimulation of PMN with aPL has no impact on induction of apoptosis. Human PMN (2×10^5^ cells per well) were incubated with LPS (100 ng/ml) with or without the addition of aPL or hIgG (10 µg/ml) Apoptotic cells were quantified by detection of DNA fragmentation via FACS. Data shown are from one representative experiment out of 3 independent with 2 replicates per group. (TIFF) [file pone.0042176.s002.tiff]
